# Green Tea Polyphenols Ameliorate the Early Renal Damage Induced by a High-Fat Diet via Ketogenesis/SIRT3 Pathway

**DOI:** 10.1155/2017/9032792

**Published:** 2017-07-26

**Authors:** Weijie Yi, Xiao Xie, Miying Du, Yongjun Bu, Nannan Wu, Hui Yang, Chong Tian, Fangyi Xu, Siyun Xiang, Piwei Zhang, Zhuo Chen, Xuezhi Zuo, Chenjiang Ying

**Affiliations:** ^1^Department of Nutrition and Food Hygiene, School of Public Health, Tongji Medical College, Huazhong University of Science and Technology, Wuhan 430030, China; ^2^MOE Key Lab of Environment and Health, School of Public Health, Tongji Medical College, Huazhong University of Science and Technology, Wuhan 430030, China; ^3^Department of Nutrition and Food Hygiene, School of Public Health and Management, Binzhou Medical University, Yantai 264003, China; ^4^Department of Hotel Management, Tourism University, Guilin 541000, China; ^5^Department of Nutrition and Food Hygiene, Xinxiang Medical University, Xinxiang 453000, China; ^6^Kecheng People's Hospital, Quzhou 324000, China; ^7^School of Nursing, Tongji Medical College, Huazhong University of Science and Technology, Wuhan 430030, China; ^8^Department of Clinical Nutrition, Tongji Hospital, Huazhong University of Science and Technology, Wuhan, Hubei 430030, China

## Abstract

**Scope:**

Several reports in the literature have suggested the renoprotective effects of ketone bodies and green tea polyphenols (GTPs). Our previous study found that GTP consumption could elevate the renal expression of the ketogenic rate-limiting enzyme, which was decreased by a high-fat diet (HFD) in rats. Here, we investigated whether ketogenesis can mediate renoprotection by GTPs against an HFD.

**Methods and Results:**

Wistar rats were fed a standard or HFD with or without GTPs for 18 weeks. The renal oxidative stress level, kidney function, renal expression, and activity levels of mitochondrial 3-hydroxy-3-methylglutaryl-CoA (HMG-CoA) synthase 2 (HMGCS2) and sirtuin 3(SIRT3) were detected. The increased renal oxidative stress and the loss of renal function induced by the HFD were ameliorated by GTPs. Renal ketogenesis and SIRT3 expression and activity levels, which were reduced by the HFD, were restored by GTPs. In vitro, HEK293 cells were transfected with the eukaryotic expression plasmid pcDNA HMGCS2. GTP treatment could upregulate HMGCS2 and SIRT3 expression. Although SIRT3 expression was not affected by HMGCS2 transfection, the 4-hydroxy-2-nonenal (4-HNE) level and the acetyl-MnSOD (K122)/MnSOD ratio were reduced in HMGCS2-transfected cells in the context of H_2_O_2_.

**Conclusion:**

The ketogenesis/SIRT3 pathway mediates the renoprotection of GTPs against the oxidative stress induced by an HFD.

## 1. Introduction

Ketogenesis is triggered during times of prolonged exercise, fasting, calorie restriction (CR) or low carbohydrate, and high lipid consumption levels. Recently, positive effects of ketogenesis have been described in terms of weight loss [1], neuroprotection [2, 3], hepatoprotection [4], and renoprotection [5–7]. Although the precise mechanisms of these effects are unclear, it is commonly considered that the promotion of energy expenditure and balance and/or reduction of reactive oxygen species (ROS) contribute to these benefits of ketogenesis.

The liver is the main, but not the only, organ to produce ketone bodies (KBs), and emerging evidence has suggested that the kidneys have a role in ketogenesis. When hepatic ketogenesis is impaired, the kidney can compensate to produce KBs to overcome starvation [8]. However, the renoprotection of KBs has become evident only in the last few years. Renal disease is an important complication in many chronic diseases, such as type 2 diabetes mellitus (T2DM) and nondiabetic obesity, which are accompanied by impaired ketogenesis or lower levels of serum KBs [9, 10]. It has been reported that ketogenesis can reverse diabetic nephropathy [6]. Additionally, an epidemiological survey found that during hyperglycemic crisis, patients with diabetic ketosis had improved kidney function and lower all-cause mortality than those without ketosis [7]. Similar to fasting and CR, treatment with *β*-hydroxybutyrate (*β*HB), the main component of KBs, could inhibit class I and class IIa histone deacetylases (HDACs) and subsequently enhance the resistance of the kidney to oxidative damage [5]. Our previous study also suggested that the increased renal expression of mitochondrial 3-hydroxy-3-methylglutaryl-CoA (HMG-CoA) synthase 2 (HMGCS2), the rate-limiting enzyme of ketogenesis, partially contributes to the antioxidant protection in the kidneys of stroke-prone, spontaneously hypertensive rats [11]. Considering all of the evidence, we proposed that promoting renal ketogenesis plays a role in ameliorating renal damage.

Epidemiological studies and animal experiments have indicated that dietary patterns and dietary components can modulate renal vascularization and function [12, 13]. A long-term high-fat diet (HFD) can lead to oxidative stress, fibrosis, and inflammation, ultimately inducing renal functional and pathological damage [13]. Additionally, obesity and metabolic abnormality, which are usually associated with high-fat intake, lead to a higher risk of renal damage [14]. In contrast, CR and phytochemicals have been verified to protect the kidney against oxidative stress [5]. Our previous studies have determined that HMGCS2 expression was increased in the liver but decreased in the kidney in the context of an HFD. However, green tea polyphenol (GTP) treatment could upregulate renal HMGCS2 expression, which is an effect similar to that of starvation [8, 15]. Hence, we presumed that in contrast to CR, the ketogenesis induced by an HFD may have tissue specificity, and the renal impairment induced by an HFD may be partially due to reduced renal ketogenesis. Additionally, similar to CR, GTP treatment could protect the kidney via promoting ketogenesis.

Mitochondrial sirtuin 3 (SIRT3), a nicotinamide adenine dinucleotide-dependent histone deacetylase, is associated with the antioxidation of CR and can improve the activity of HMGCS2 via deacetylation. KBs were verified to elevate the NAD+/NADH ratio in neurons, subsequently increasing the antioxidant activity of SIRT3 [16]. However, whether a similar mechanism exists in the kidney was not known. In fact, SIRT3 is abundantly expressed in the kidney and can protect proximal tubular cells from the lipotoxicity induced by palmitate by enhancing the mitochondrial oxidative capacity and antioxidant defense [17]. Additionally, GTPs and epigallocatechin gallate (EGCG, the principal component of GTPs) were reported to increase SIRT3 expression [18]. Based on the above facts, we hypothesized that SIRT3 might participate in the renoprotection of ketogenesis. Therefore, the present study investigated the renoprotection of GTPs against an HFD and the roles of ketogenesis/SIRT3 in these processes.

## 2. Materials and Methods

### 2.1. Animal Treatments

Forty male Wistar rats weighing 160–180 g were fed standard. After one week of acclimation, all of the animals were randomly divided into four groups (CON, CON + GTPs, HFD, and HFD + GTPs). The CON group (*n* = 10) was fed standard as the control. The CON + GTPs group (*n* = 10) was fed standard, to which GTPs were added based on the food intake and weight (200 mg/kg bw). The HFD group was fed a modified HFD (60% standard diet, 12% lard, 10% sugar, 8% yolk powder, 6% peanut powder, 3% casein, and 1% milk powder, *w*/*w*). Additionally, the HFD + GTPs group was treated with 200 mg/kg bw GTPs, which was added in the HFD, based on the food intake and weight. For rats on the standard diet, the caloric content (3.33 kcal/g) was derived from diet containing 24.02% protein, 13.51% fat, and 62.46% carbohydrate by energy. For rats on the HFD, the caloric content (4.54 kcal/g) was derived from diet containing 16.72% protein, 45.14% fat, and 38.14% carbohydrate by energy. Rats were housed with a 12-hour light-dark cycle. Body weight (every week) and food intake (every day) were recorded regularly. In the 16th week, the animals were subjected to an intraperitoneal glucose tolerance test (IPGTT) or an insulin tolerance test (ITT) after 12 or 6 hours of fasting. In weeks 17 and 18, all of the animals were housed individually in metabolic cages to collect 24-hour urine. Forty rats were decapitated in the 18th week, and the kidneys were isolated and snap-frozen in liquid nitrogen and then stored at −80°C until further use. Part of the kidney was fixed and embedded in paraffin.

### 2.2. Cell Culture and Treatments

Human embryonic kidney 293 (HEK 293) cells were obtained from the Cell Bank of the Chinese Academy of Sciences (Shanghai, China). HEK293 cells were cultured in Dulbecco's modified Eagle's medium (DMEM)/high glucose containing 10% fetal bovine serum and supplemented with penicillin/streptomycin in 5% CO_2_ at 37°C. The plasmids were purchased from Vigene Biosciences. After extraction and verification, plasmid pcDNA HMGCS2 transfection was conducted using Lipofectamine 3000 transfection reagent according to the manufacturer's instructions. The transfected cells were treated with GTPs (4 *μ*g/ml, 24 hours) or H_2_O_2_ (0.1 mM, 6 hours) separately.

### 2.3. Reagents

GTPs (91.21% catechins and 71.72% EGCG) were purchased from Corona Science & Technology Development Co. Ltd. (Fu Zhou, China). Rabbit polyclonal antibodies (anti-MnSOD, anti-HMGCS2, anti-Nampt, and anti-catalase) were purchased from Santa Cruz Biotechnology Inc. (Santa Cruz, USA). Anti-FOXO3a and anti-SIRT3 antibodies were from Cell Signaling Technology Inc. (Danvers, USA). Anti-SOD2/MnSOD (acetyl K122) and anti-4-HNE antibodies were from Abcam Inc. (Cambridge, UK). Total cholesterol (TC), triglycerides (TG), high-density lipoprotein-cholesterol (HDL-C), low-density lipoprotein-cholesterol (LDL-C), and blood glucose were detected using kits from BioSino Bio-Technology & Science Inc. (Beijing, China). ELISA kits for the quantitative measurement of serum cystatin C and insulin were from Biovendor Inc. (Heidelberg, Germany) and Mercodia AB (Uppsala, Sweden). Creatinine and N-acetyl-*β*-D-glucosaminidase (NAG) assay kits were purchased from the Nanjing Jiancheng Bioengineering Institute (Jiangsu, China). DMEM/high glucose was purchased from Gibco Inc. (New York, USA). Lipofectamine 3000 transfection reagent was from Invitrogen Inc. (California, USA).

### 2.4. Intraperitoneal Glucose Tolerance Test and Insulin Tolerance Test

For the IPGTT, rats were fasted for 12 hours and subsequently received an intraperitoneal injection of glucose (2 g/kg body weight). Blood glucose levels were measured at 0, 15, 30, 60, and 120 minutes after injection using a glucometer (ACCU-CHEK Performa, Roche). For the ITT, the rats were injected intraperitoneally with recombinant human regular insulin (0.8 U/kg body weight) after fasting for 6 hours. Blood glucose concentrations were monitored at 0, 30, 60, 90, and 120 minutes after injection, still using a glucometer. The area under the curve was calculated by trapezoidal summation.

### 2.5. Serum Biochemistry Analysis

Blood samples were collected after standing for 30 minutes at room temperature and were then centrifuged at 3000 rpm for 10 minutes to separate the serum. Serum glucose, TG, TC, LDL-C, HDL-C, and insulin were analyzed using commercial kits according to the manufacturers' protocols.

### 2.6. Histology and Immunohistochemistry

Briefly, renal paraffin sections, approximately 3-4 *μ*m in thickness, were stained with hematoxylin-eosin after being deparaffinized and rehydrated. For immunohistochemical staining of the kidney, deparaffinized and rehydrated sections were blocked with bovine serum albumin (BSA) after antigen retrieval and blocking endogenous peroxidase and were then incubated with 4-HNE antibody at 4°C overnight. After incubation with horseradish peroxidase- (HRP-) labeled goat-anti-rabbit secondary antibody, the sections were stained with 3,3′-diaminobenzidine (DAB). The mean optical density was analyzed using Image-Pro Plus software.

### 2.7. Assessment of Renal Function

In weeks 17 and 18, all of the animals were housed individually in metabolic cages to collect 24-hour urine samples. The samples were centrifuged at 3000 ×g for 10 minutes to remove suspended particles and were then stored in aliquots at −80°C. The creatinine levels, activity of urinary NAG, and serum cystatin C were measured using enzymatic assays. The urinary microalbumin levels were measured using a BioSystems A25 analyzer (BioSystems, Spain). The creatinine clearance rate (Ccr) was calculated using the following formula: Ccr (ml/h/100 g of body weight) = [urinary creatinine (mg/dl) × urine volume (ml/h)]/[serum creatinine (mg/dl) × body weight (g)/100].

### 2.8. Immunoblotting Analysis

Kidney cortex tissues were lysed with radioimmunoprecipitation assay (RIPA) buffer (Beyotime, Shanghai, China), and proteins were quantified using the bicinchoninic acid (BCA) method (Beyotime, Shanghai, China). Protein lysates (10–50 *μ*g) were electrophoresed on SDS-polyacrylamide gels and were electrotransferred to polyvinylidene difluoride (PVDF) membranes (Millipore, Billerica, MA, USA). After blocking for 1 h with 10% skim milk, the membranes were washed and incubated with primary antibody overnight at 4°C, followed by incubation with HRP-conjugated second antibody for 1 h at room temperature. The proteins were then visualized using the ECL Western blotting detection reagents (Millipore, Billerica, MA, USA). GAPDH or *β*-actin served as an internal control.

### 2.9. MnSOD, CAT, Total SOD, and Cu/Zn SOD Activity Levels and MDA, NAD, and *β*-Hydroxybutyrate Levels in the Kidney Cortex and Serum

The MnSOD, CAT, total SOD, and Cu/Zn SOD activity and MDA levels in the kidney cortex were analyzed using commercial kits according to the manufacturers' instructions (Nanjing Jiancheng Bioengineering Institute, Jiangsu, China).

The NAD (BioVision, San Francisco, USA) and *β*-hydroxybutyrate (Cayman, Michigan, USA) levels in the kidney cortex and serum were detected using commercial kits according to the manufacturers' instructions.

### 2.10. Statistical Analysis

Treatment effects were analyzed using an independent one-way ANOVA with a least significant difference (LSD) post hoc test (SPSS 12.0). Descriptive statistics are presented as the mean ± SD. *P* < 0.05 was considered to be statistically significant. GraphPad Prism 6.0 software was used to perform all statistical analyses.

### 2.11. Ethics Statement

All animal protocols and procedures conformed to the guidelines and authorization for the use of laboratory animals and were approved by the Committee on the Ethics of Animal Experiments of the Huazhong University of Science and Technology (permit number: S412).

## 3. Results

### 3.1. Effects of GTPs on the Body Weight and Blood Biochemical Indices

After 18 weeks of feeding, the body weights and visceral fat masses of rats in the HFD group had increased markedly (*P* < 0.05). GTPs could reduce these parameters without affecting the energy intake (*P* < 0.05). No significant difference was observed in kidney weight between the CON and HFD groups. Nevertheless, the kidney coefficient of the HFD group was inferior to that of the CON group (*P* < 0.05). Adding GTPs to the control diet had no effect on these parameters (Table 1).

Sera from rats in the HFD group had high TC and LDL-C and low HDL-C (*P* < 0.05). However, the blood glucose and TG levels were the same as those of the CON group. Decreased levels of blood glucose, TG, TC, and LDL-C and higher levels of HDL-C were observed in the HFD + GTPs group (*P* < 0.05). Additionally, the data indicated that GTP treatment could reduce the serum LDL-C level of the CON group (*P* < 0.05) (Table 1).

### 3.2. Effects of GTPs on the IPGTT and the ITT of Rats

**Figure 1:**
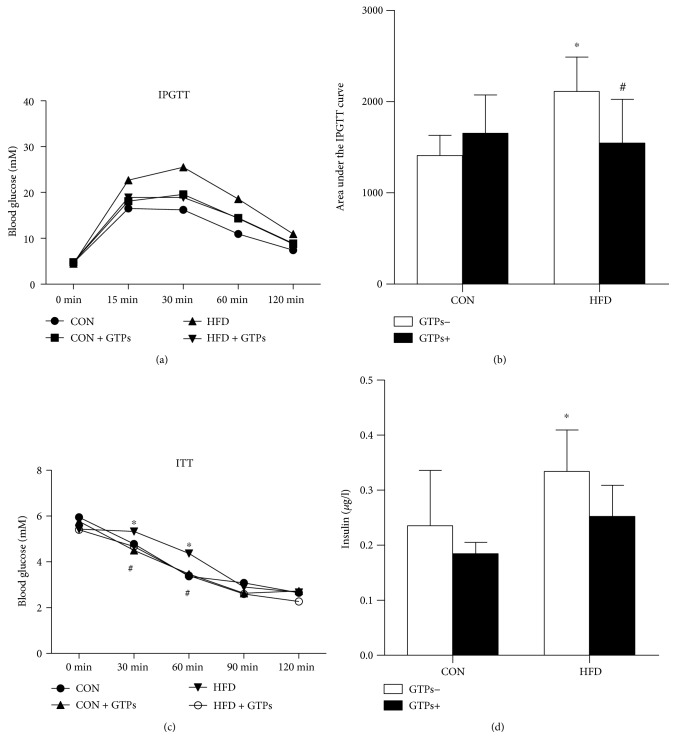
Effects of GTPs and HFD on insulin resistance. (a, b) Results of the IPGTT. (c) Results of the ITT. (d) Serum insulin concentrations of rats in the different groups. The data are represented as the mean ± SD ((a), (b), and (d), *n* = 5; (c), *n* = 3); ^∗^*P* < 0.05 versus the control group and ^#^*P* < 0.05 versus the HFD group.

Insulin resistance is closely related to chronic kidney disease (CKD), and the insulin level can disturb ketogenic activities. Thus, we measured the sensitivity and serum concentration of insulin. The area under the IPGTT curve was larger in the HFD group than in the vehicle group (*P* < 0.05) (Figures 1(a) and 1(b)). Additionally, after intraperitoneal insulin injection, the serum glucose levels at 30 and 60 minutes were much higher in the HFD group than in the CON group (Figure 1(c)). The HFD increased the serum insulin concentration, which appeared to be reduced by GTP treatment, though the difference was not statistically significant (Figure 1(d)).

### 3.3. GTPs Ameliorate the Impairment of Renal Function Induced by HFD

**Figure 2:**
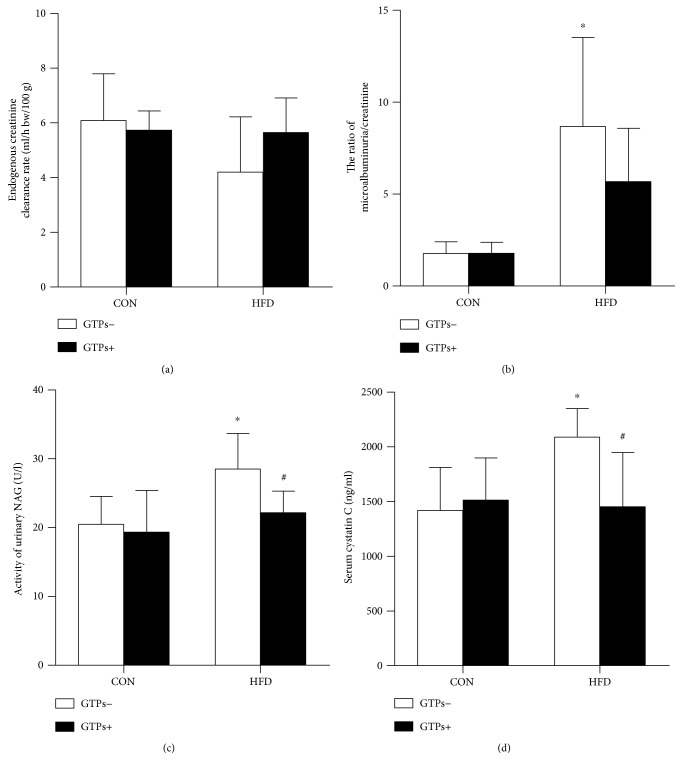
Effects of GTPs and HFD on kidney function. (a) Endogenous Ccr. (b) Urine microalbuminuria/creatinine ratio. (c) Activity of urinary NAG. (d) Serum cystatin C level. The data are represented as the mean ± SD (*n* = 6–8); ^∗^*P* < 0.05 versus the control group and ^#^*P* < 0.05 versus the HFD group.

We speculated that an HFD could lead to renal pathological changes; however, no differences were observed among the four groups according to hematoxylin and eosin (HE) staining. The Ccr, serum cystatin C level, urinary NAG activity, and microalbuminuria/creatinine ratio were detected to reflect kidney injury. Ccr is a regular indicator to assess kidney function, and the latter three are used to detect early injury to the kidney. Notable differences between the CON and HFD groups were observed in the microalbuminuria/creatinine ratio (Figure 2(b)), the urinary NAG activity (Figure 2(c)), and the serum cystatin C level (Figure 2(d)). The addition of GTPs could normalize urinary NAG activity and the serum cystatin C level, which were increased by the HFD.

### 3.4. GTPs Ameliorate the Oxidative Stress of the Renal Cortex

**Figure 3:**
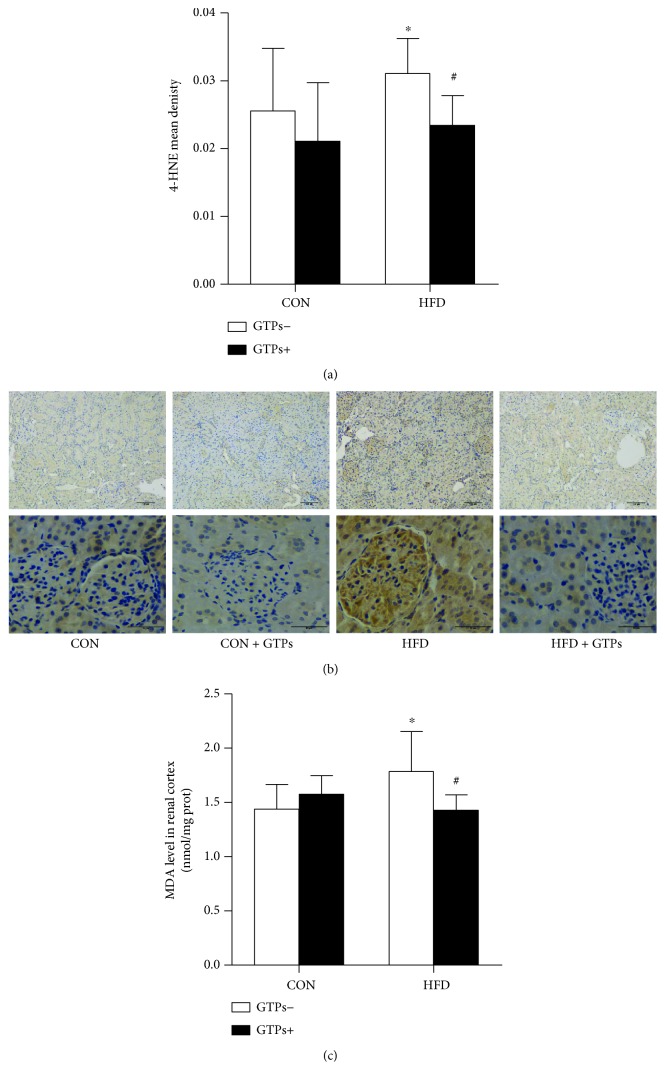
Effects of GTPs and HFD on renal cortex oxidative damage. (a, b) Immunohistochemical staining (original magnifications: 100x and 400x) and analysis of 4-HNE in the rat kidney. (c) MDA level in the rat kidney. The data are represented as the mean ± SD ((a) *n* = 3; (c) *n* = 9); ^∗^*P* < 0.05 versus the control group and ^#^*P* < 0.05 versus the HFD group.

Because of the key role of ROS in the progression of chronic kidney disease, 4-hydroxy-2-nonenal (4-HNE) and MDA were used to indicate the oxidative stress level. The 4-HNE and MDA levels in the renal cortex were dramatically increased by the HFD, while both indices could be returned to normal levels by GTP treatment (Figure 3).

### 3.5. GTPs Upregulate the Expression of HMGCS2 and Its Catalyst in the Renal Cortexes of HFD-Treated Rats

**Figure 4:**
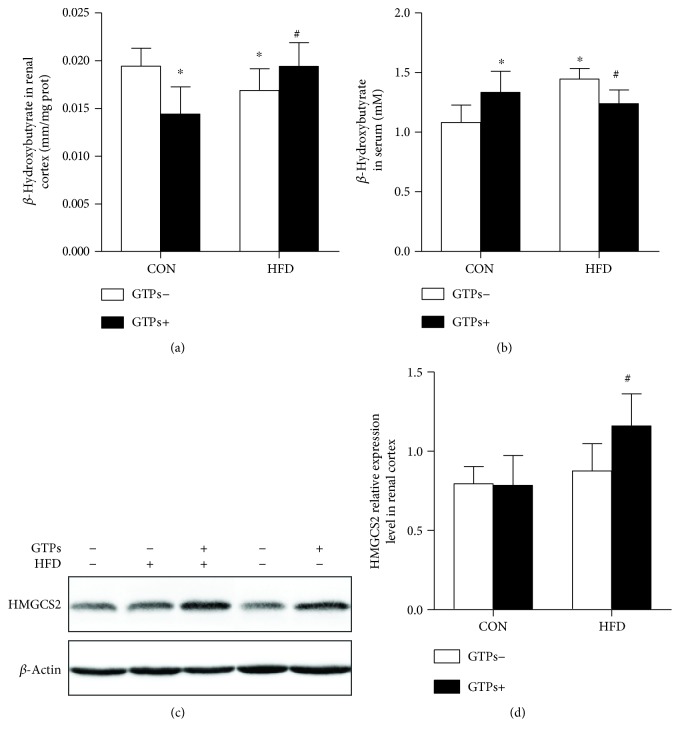
Effects of GTPs and HFD on the *β*-hydroxybutyrate concentration and the expression of a ketogenic rate-limiting enzyme. (a, b) *β*-Hydroxybutyrate concentrations in the kidney and serum. (c, d) Western blot and density analyses of renal HMGCS2 protein expression. The data are represented as the mean ± SD (*n* = 5–9); ^∗^*P* < 0.05 versus the control group and ^#^*P* < 0.05 versus the HFD group.

To analyze whether the oxidative stress change was associated with ketogenesis, we detected the protein expression levels of the control enzyme of ketogenesis (HMGCS2) by Western blotting and its catalyst (*β*-hydroxybutyrate) in the renal cortex. Additionally, we detected the serum *β*-hydroxybutyrate concentration to indicate the ketogenic activity of the liver. Compared with the control group, in the CON + GTPs and HFD groups, *β*-hydroxybutyrate was reduced in the kidney and boosted in the serum (Figures 4(a) and 4(b)). However, in the context of an HFD, GTP treatment could significantly upregulate renal HMGCS2 protein expression (Figures 4(c) and 4(d)) and its production (Figure 4(a)) but decrease serum *β*-hydroxybutyrate (Figure 4(b)).

### 3.6. GTPs Have No Effect on MnSOD, CAT, and FOXO3a Protein Expression but Can Increase Antioxidant Activity Levels in the Context of HFD

**Figure 5:**
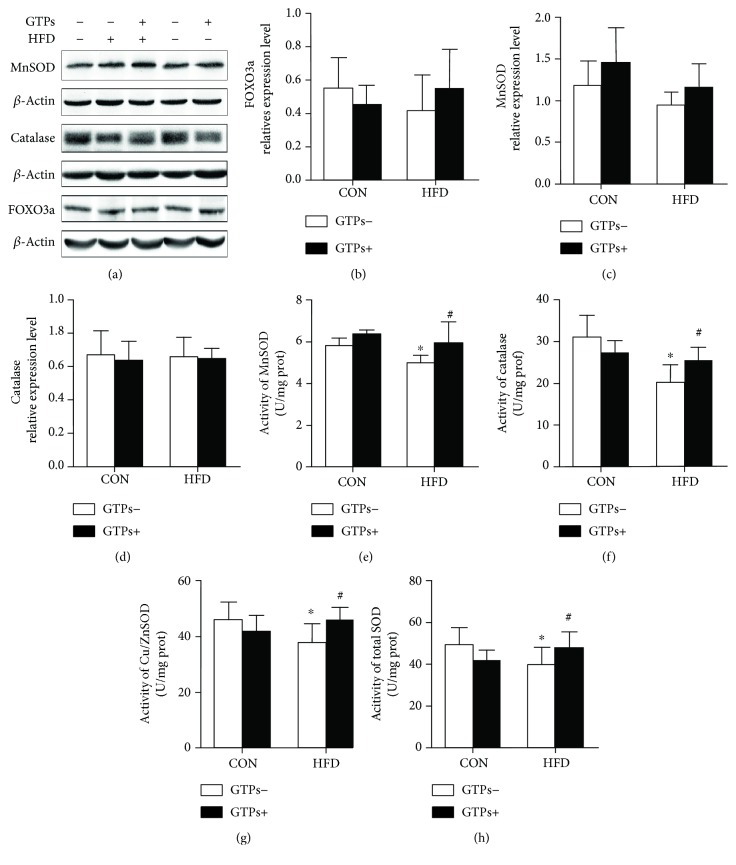
Effects of GTPs and HFD on renal antioxidant-related protein expression and antioxidant activities. (a) Western blot bands of renal MnSOD, CAT, and FOXO3a. (b, c, d) Relative expression levels of renal MnSOD, CAT, and FOXO3a. (e–h) Activities of MnSOD, CAT, Cu/ZnSOD, and total SOD. The data are represented as the mean ± SD (*n* = 5–8); ^∗^*P* < 0.05 versus the control group and ^#^*P* < 0.05 versus the HFD group.

It has been reported that *β*-hydroxybutyrate supplementation could upregulate the expression levels of FOXO3a, MnSOD, and other antioxidants. However, in the present study, the MnSOD protein expression level was increased in GTP-treated rats and decreased in HFD-treated rats, though no significant difference was detected among the four groups. Similarly, GTPs and the HFD had no effect on CAT and FOXO3a protein expression (Figures 5(a), 5(b), 5(c), and 5(d)). However, the activities of MnSOD, CAT, Cu/ZnSOD, and total SOD in the rat renal cortex were lower in the HFD group than in the CON group. Additionally, the activities were increased significantly in the HFD + GTPs group (Figures 5(e), 5(f), 5(g), and 5(h)).

### 3.7. GTPs Restore SIRT3 Protein Expression and Activity Levels in the Renal Cortexes of HFD-Treated Rats

**Figure 6:**
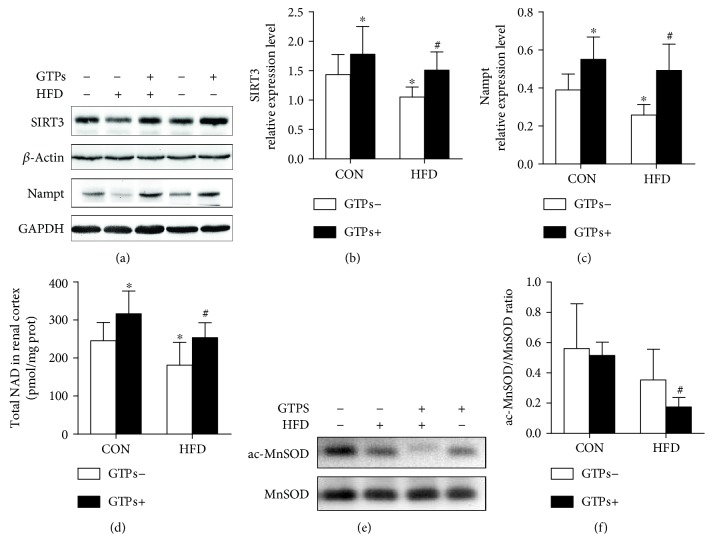
Effects of GTPs and HFD on SIRT3 expression and activity. (a, b, c) Western blot bands and relative expression levels of renal SIRT3 and Nampt. (e, f) Western blot analysis of acetylated MnSOD and total MnSOD and the ratio of acetylated MnSOD/MnSOD. The data are represented as the mean ± SD (*n* = 6–8); ^∗^*P* < 0.05 versus the control group and ^#^*P* < 0.05 versus the HFD group.

Accompanied by the generation of *β*-hydroxybutyrate, the mitochondrial ratio of NAD+/NADH was changed. Additionally, SIRT3, an NAD+-dependent histone deacetylase, is also located in the mitochondrion and can exert antioxidative effects. Furthermore, HMGCS2 activity can be upregulated by SIRT3.Therefore, to confirm whether SIRT3 was involved in the process of ketogenesis against oxidative stress, we detected SIRT3 protein expression. Additionally, SIRT3 activity was indicated by ac-MnSOD (K122) and of MnSOD activity. Nicotinamide phosphoribosyltransferase (Nampt) protein expression and the NAD level in the cortex could also partially reflect SIRT3 activity. With or without an HFD, supplementation with GTPs could elevate renal SIRT3 and Nampt protein expression (Figures 6(a), 6(b), 6(c), and 6(d)). The two groups treated with GTPs showed higher levels of MnSOD deacetylation than the other two groups, but only the difference between the HFD and HFD + GTPs groups was statistically significant (Figures 6(e) and 6(f)). Additionally, these data were consistent with MnSOD activity (Figure 5(e)) and NAD levels (Figure 6(d)).

### 3.8. HEK293 Cells Transfected with the Plasmid pcDNA *HMGCS2* Show Lower Levels of 4-HNE and Acetylated MnSOD

**Figure 7:**
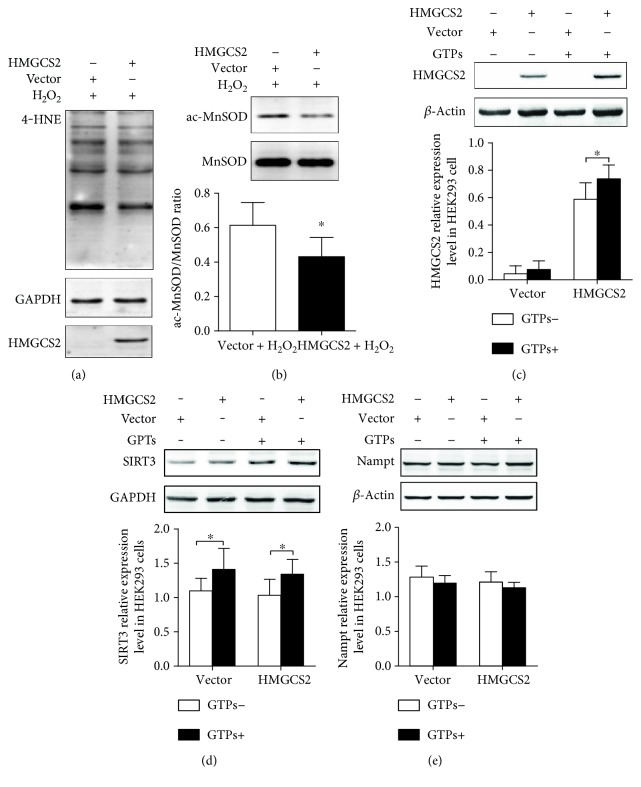
Transfection of HMGCS2 reduces the levels of acetylated MnSOD (K122) and 4-HNE. (a) 4-HNE levels in transfected cells treated with 0.1 mM H_2_O_2_. (b) Western blot analysis of acetylated MnSOD (K122) and total MnSOD and the ratio of acetylated MnSOD/MnSOD. (c, d, e) HMGCS2, SIRT3, and Nampt protein expression in transfected HEK293 cells treated with GTPs. The data are represented as the mean ± SD (*n* = 4–6); ^∗^*P* < 0.05.

To confirm that ketogenesis could play a role in antioxidation, HEK293 cells transfected with HMGCS2 were treated with 0.1 mM H_2_O_2_. The level of 4-HNE and the ratio of acetylated-MnSOD (K122)/MnSOD were reduced by transfection (Figures 7(a) and 7(b)). Additionally, the HMGCS2 and SIRT3 protein expression levels in HEK293 cells transfected with HMGCS2 could be upregulated by GTP treatment (Figures 7(c) and 7(d)). Moreover, SIRT3 expression was not affected by HMGCS2 transfection (Figure 7(d)). Neither GTP treatment nor HMGCS2 transfection could influence Nampt expression (Figure 7(e)).

## 4. Discussion

The antioxidative capacity of GTPs is mostly attributed to their capacity to scavenge free radicals, chelate metal ions, provoke antioxidant enzymes, and inhibit redox-sensitive transcription. To the best of our knowledge, no one has reported on the role of ketogenesis in the antioxidation of GTPs. In the current study, we found that oral supplementation with GTPs can reduce renal oxidative stress induced by an HFD through the ketogenesis/SIRT3 pathway.

Excess fat intake induced by an HFD can lead to renal lipotoxicity, which is directly reflected in dyslipidemia, insulin resistance, lipid deposition, higher inflammation, and the oxidative stress level, with subsequent functional and pathological renal damage [19]. Although the initial event in lipid-induced kidney damage has not been defined, oxidative stress is recognized as one of the most significant pathogenic mechanisms. The inhibition of lipid deposition [20–22] and decreases in ROS and inflammation levels [23–25] could improve impaired kidney function. In the current study, in addition to increased body weight, insulin resistance, and dyslipidemia, the rats fed with the HFD showed higher oxidative stress levels and impaired renal function, which were reflected by biomarkers of early renal impairment, including serum cystatin C, urine NAG activity, microalbuminuria, and Ccr [26–28]. GTP administration could ameliorate these changes induced by the HFD, supporting the antioxidative and renoprotective roles of GTPs. Additionally, the renal *β*HB concentration was decreased in the HFD-treated group and increased in the GTP-supplemented group, although the serum *β*HB concentrations were higher in the HFD-treated group. These were consistent with our previous study that the expression of HMGCS2 could be upregulated by GTPs through PPAR-*α* pathway. An inverse relationship was detected between the renal *β*HB concentration/HMGCS2 expression and oxidative stress. Emerging evidence has supported the idea that KBs, especially *β*HB, can exert health-protective effects beyond serving as an energy source. KBs or a ketogenic diet can be used to resist oxidation and inflammation [16, 29–33], control blood glucose in diabetic individuals [34], inhibit tumor growth [35], mediate signaling pathways [36], and stimulate autophagy [37]. Most KBs are synthesized in the liver. However, the kidneys can compensate to produce KBs to overcome starvation when hepatic ketogenesis is impaired [8]. Additionally, KB/ketogenesis was reported to protect kidney function against oxidative and other stresses [6, 7, 38]. Furthermore, *β*HB was demonstrated to protect the kidney from ROS through inhibiting the activities of class I and class IIa histone deacetylases, subsequently increasing the expression of FOXO3a and its well-defined targets MnSOD and catalase, which contributed to its protective activity against oxidative stress [5]. Combining these findings with ours, we inferred that the increased ketogenesis may contribute to the antioxidation of GTPs. Therefore, the antioxidation of ketogenesis was detected using HMGCS2-transfected HEK293 cells in vitro. GTP treatment could upregulate HMGCS2 protein expression, and the 4-HNE level was decreased in HMGCS2-transfected cells in the context of H_2_O_2_. These data indicated that GTPs could upregulate renal ketogenesis reduced by an HFD and that ketogenesis contributed to the antioxidation.

In contrast to the effects on the kidney, hepatic ketogenesis was increased by HFD intake, as indicated by the elevated concentration of serum *β*HB [39]. However, during CR or fasting, ketogenesis is increased in both the liver and the kidney [8], and we demonstrated this increase in another study (Supplementary Figure 1 available online at https://doi.org/10.1155/2017/9032792). These findings might, to some extent, explain why CR and HFDs lead to distinct effects on the kidney, although both diets can elevate serum KB concentrations. Furthermore, the importance of ketogenesis to the kidney in the context of an HFD is suggested.

It has been reported that HMGCS2 can be deacetylated by SIRT3, a member of the class III histone deacetylases (sirtuins), subsequently increasing ketogenesis [40, 41]. Thus, the reduced SIRT3 expression in the HFD-treated group can explain why the renal KB concentration was decreased despite their being no difference in HMGCS2 expression compared with the CON group. In fact, SIRT3 is abundantly expressed in renal mitochondria and plays crucial roles in mitochondrial respiration, fatty acid *β*-oxidation, energy production, antioxidant activity, and deacetylation [42, 43]. Our previous study indicated that GTP treatment could reduce renal oxidative stress through PPAR-*α*/SIRT3 pathway [15]. And increased SIRT3 expression and activity levels could reduce renal injury and improve kidney function [17, 44]. In contrast, *sirt3*-deficient mice displayed much more severe acute kidney injury and died [44]. We also observed improved renal function in GTP-treated rats, which showed high SIRT3 expression. Nevertheless, according to the in vitro results, this increase in SIRT3 was independent of HMGCS2.

Ketogenesis/KBs can also disturb the NAD+/NADH ratio, which can affect SIRT3 activity [16, 29, 45]. The reduction in ROS plays an important role in SIRT3-mediated antioxidation. However, this reduction is blunted in *MnSOD* knockout mouse embryonic fibroblasts [46]. Nevertheless, without SIRT3, only 10% of ROS was reduced in the cellular overexpression of MnSOD alone [46] because the latter, a major mitochondrial antioxidant enzyme, can be activated by SIRT3 via deacetylation at lysine 122 site and can mediate SIRT3 antioxidation [46, 47]. The attenuated acetylation of renal MnSOD (K122) in the HFD + GTPs group suggested increased MnSOD and SIRT3 activity levels [47], findings that were consistent with MnSOD activity levels. MnSOD and CAT expression levels were also reported to be upregulated by SIRT3 [48]. We only observed increased enzymatic activity in the HFD + GTPs group. Although there was no effect on SIRT3 expression in vitro, the *HMGCS2*-transfected group showed a decreased 4-HNE level and acetyl-MnSOD (K122)/MnSOD ratio after treatment with H_2_O_2_. These data indicate that HMGCS2 could increase SIRT3 activity against oxidative stress. When ketogenesis is initiated, the acetoacetate produced needs to be transformed into *β*HB in the mitochondria. Accompanying this process, the mitochondrial NAD+/NADH ratio increases [16, 29, 45], subsequently activating SIRT3. This process might be a mechanism by which HMGCS2 upregulates SIRT3 activity. The renal ketogenesis and SIRT3 activity can also be influenced by the expression of Nampt, the rate-limiting enzyme that catalyzes NAD+ biosynthesis in mammals [38, 49]. In the present study, increased Nampt protein expression upon GTP treatment was only observed in vivo and could not be verified in vitro. After all, the environment in vivo is much more complicated than that in vitro. And the duration of intervention also could influence the effect. In an animal model, the kidney was continually exposed to GTPs, while it was only 24 hours for the HEK293 cells to expose to GTPs. Taken together, in the context of an HFD, the increased SIRT3 activity following GTP supplementation was due to increased renal ketogenesis. Nevertheless, in the future, it will be necessary to demonstrate the role of ketogenesis in the kidney with renal *HMGCS2* knockdown animals.

## 5. Conclusion

In the current study, we demonstrated that in the context of an HFD, renal ketogenesis could resist oxidation via SIRT3 activation, indicating a role for ketogenesis in defending renal impairment induced by a Western diet. Additionally, we found that GTPs could provoke renal ketogenesis, suggesting a new possible protective mechanism for phytochemicals. Improving KB production by GTP supplementation instead of ketogenic diet consumption or CR will avoid various side effects, be best for long-term adherence, and provide clues for the prevention of related diseases. In conclusion, this study sheds light on the possible renoprotective role of GTPs against the HFD via antioxidation, which is mediated by ketogenesis/SIRT3.

## Supplementary Material

Supplementary Figure 1. Effects of CR on renal ketogenesis. CR group were treated with 30% calorie restriction of CON. CR+GTPs group were treated with 30% calorie restriction of CON with 200mg/kg.bw GTPs. (A) Western blot analysis of HMGCS2, SIRT3, ac-MnSOD, MnSOD and Nampt expression levels in the renal cortex. (B-E) Density analysis of renal HMGCS2, SIRT3, ac-MnSOD/MnSOD and Nampt. (F,G) β-hydroxybutyrate concentrations in the kidney and serum. (H) Total NAD level in the renal cortex. The data are represented as the mean±SD (n=5-6); ∗P<0.05 vs the control group and &P <0.05 vs the CR group.

## Figures and Tables

**Table 1:** Effects of GTPs on weight and blood biochemical indices in different rat groups (*χ* ± *s*, *n* ≥ 5).

	CON	CON + GTPs	HFD	HFD + GTPs
Initial weight (g)	195.00 ± 8.17	192.50 ± 10.51	193.80 ± 7.51	192.80 ± 12.60
Final weight (g)	530.80 ± 16.65	527.10 ± 24.50	606.30 ± 24.18^∗^	562.40 ± 55.02^#^
Food intake (g/day)	27.05 ± 1.84	27.66 ± 1.68	20.62 ± 1.09^∗^	20.96 ± 0.94
Energy intake (kcal/day)	89.30 ± 6.28	91.61 ± 5.68	92.52 ± 5.12	94.07 ± 4.26
Visceral fat mass (g)	13.57 ± 0.89	16.42 ± 4.82	29.41 ± 8.66^∗^	20.99 ± 6.43^#^
Visceral fat coefficient	2.62 ± 0.25	3.14 ± 0.78	5.00 ± 1.24^∗^	3.82 ± 0.99^#^
Kidney weight (g)	3.27 ± 0.53	3.22 ± 0.39	3.29 ± 0.33	2.98 ± 0.17
Kidney coefficient	0.63 ± 0.07	0.62 ± 0.06	0.56 ± 0.06^∗^	0.55 ± 0.04
Blood glucose (mmol/l)	6.24 ± 0.52	6.24 ± 0.69	6.56 ± 0.58	5.57 ± 0.34^#^
Triglyceride (mmol/l)	0.93 ± 0.37	0.74 ± 0.07	1.22 ± 0.22	0.92 ± 0.24^#^
Total cholesterol (mmol/l)	1.63 ± 0.16	1.59 ± 0.35	1.99 ± 0.26^∗^	1.49 ± 0.14^#^
HDL-C (mmol/l)	1.34 ± 0.28	1.30 ± 0.19	0.84 ± 0.26^∗^	1.43 ± 0.14^#^
LDL-C (mmol/l)	0.29 ± 0.12	0.13 ± 0.08^∗^	0.68 ± 0.11^∗^	0.23 ± 0.12^#^

Some rats were fed a control diet with or without GTPs for 18 weeks, and others were fed an HFD with or without GTPs for 18 weeks. ^∗^*P* < 0.05 versus the control group and ^#^*P* < 0.05 versus the HFD group.

## Abbreviations

HMGCS2: Mitochondrial 3-hydroxy-3-methylglutaryl-CoA (HMG-CoA) synthase 2
*β*HB: *β*-Hydroxybutyrate
HDAC: Histone deacetylase
SIRT3: Sirtuin 3
MnSOD: Manganese superoxide dismutase
FOXO3a: Forkhead box O3a
GTPs: Green tea polyphenols
CON: Control
HFD: High-fat diet
Nampt: Nicotinamide phosphoribosyltransferase
4-HNE: 4-Hydroxy-2-nonenal
KB: Ketone body
CR: Calorie restriction
ROS: Reactive oxygen species
NAG: N-Acetyl-*β*-D-glucosaminidase
TC: Total cholesterol
TG: Triglycerides
HDL-C: High-density lipoprotein-cholesterol
LDL-C: Low-density lipoprotein-cholesterol.
