# Supplementary figures and images for: Green Tea Polyphenols Ameliorate the Early Renal Damage Induced by a High-Fat Diet via Ketogenesis/SIRT3 Pathway
Source: Oxid Med Cell Longev. 2017 Jul 26;2017:9032792. doi: 10.1155/2017/9032792 (PMC5549484; doi:10.1155/2017/9032792)

## Slide 1
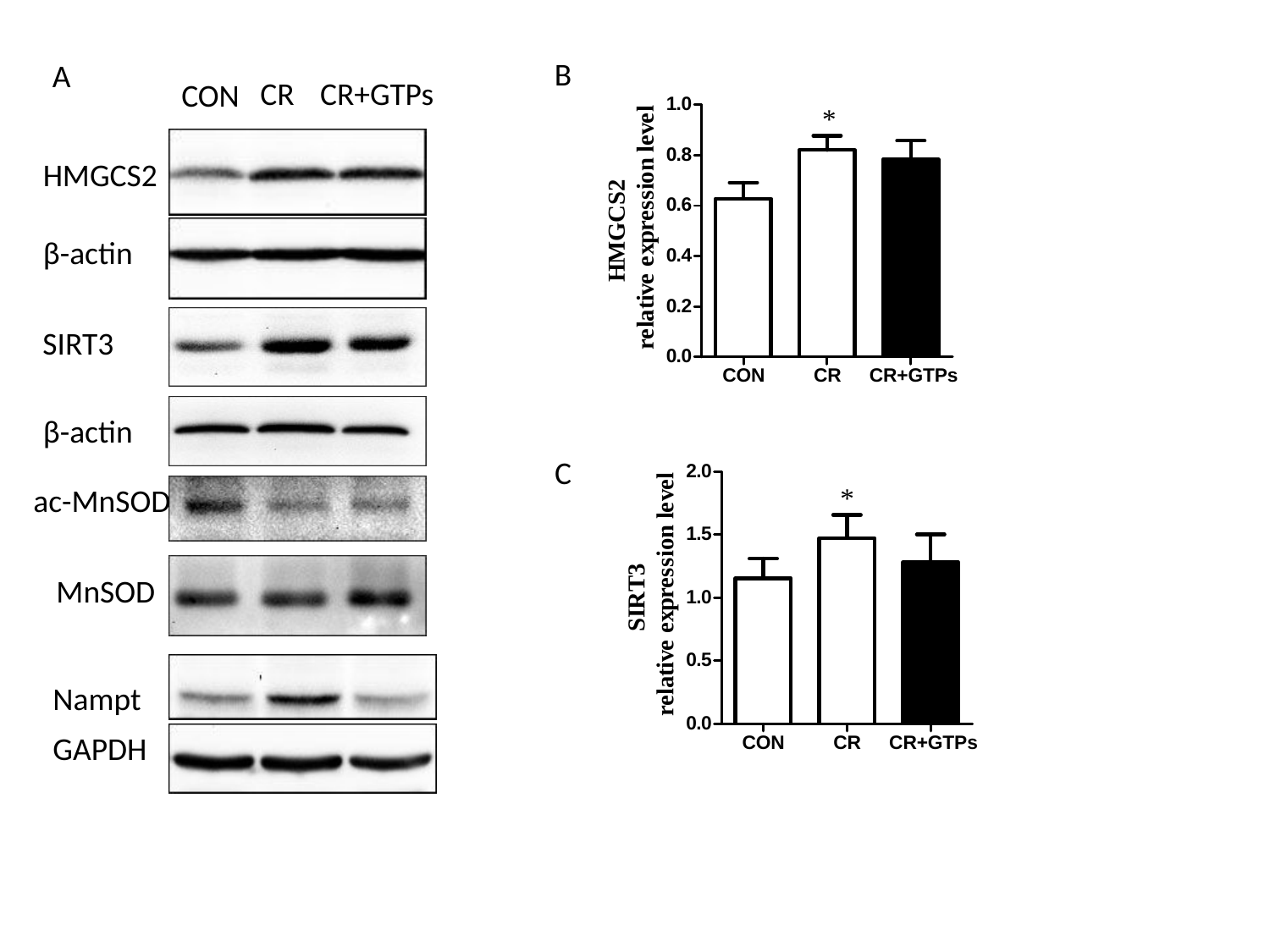

B
A
CR
CR+GTPs
CON
HMGCS2
β-actin
SIRT3
β-actin
C
ac-MnSOD
MnSOD
Nampt
GAPDH

## Slide 2
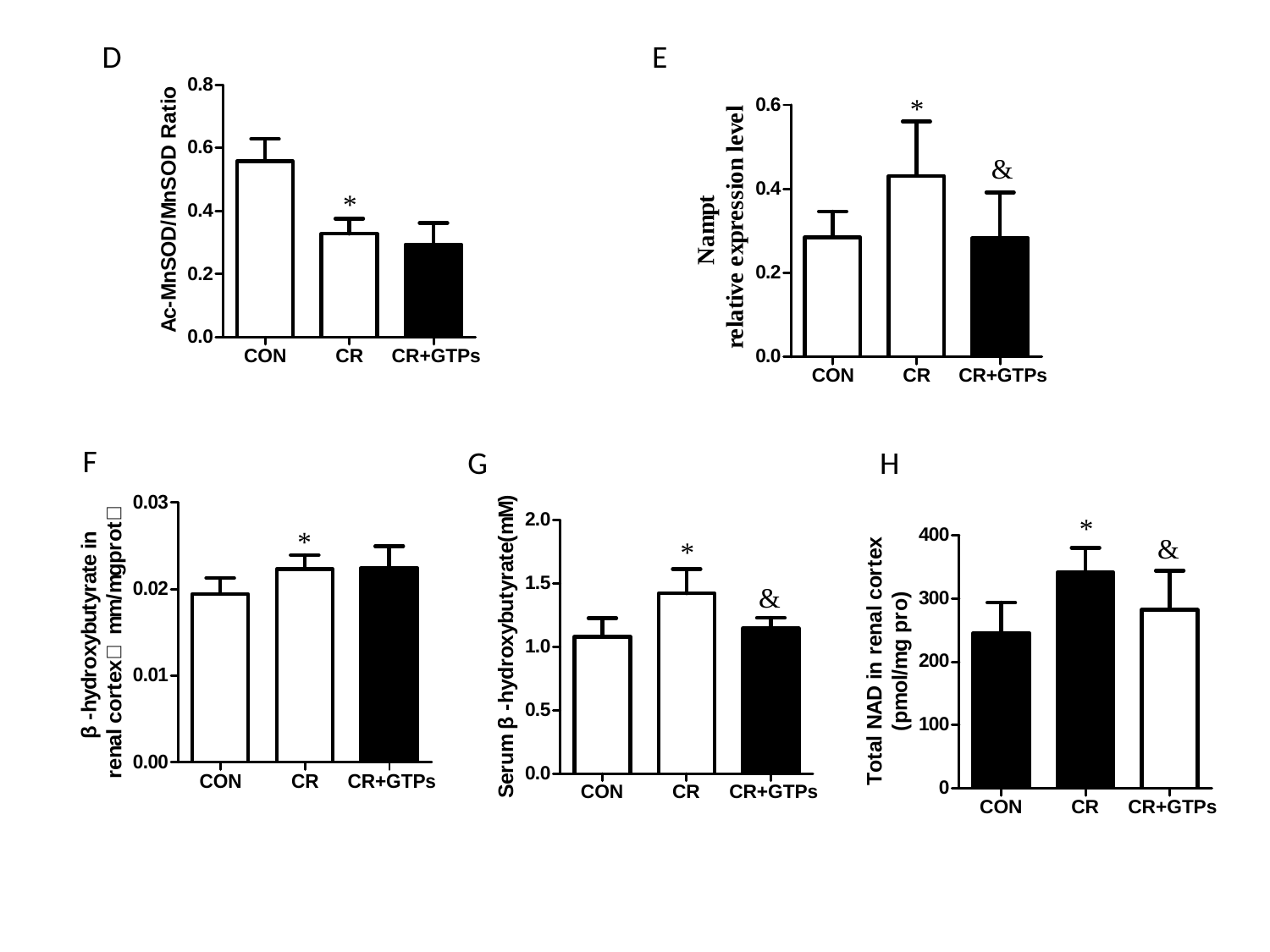

D
E
F
G
H

Supplement: Supplementary file 2 [file 9032792.f2.pptx]
